# Supplementary material for: GMPR: A robust normalization method for zero-inflated count data with application to microbiome sequencing data
Source: PeerJ. 2018 Apr 2;6:e4600. doi: 10.7717/peerj.4600 (PMC5885979; doi:10.7717/peerj.4600)
Supplement: Supplemental Information 1 [file peerj-06-4600-s001.pdf]

GMPR: A robust normalization method for zero-inflated count data with application to microbiome sequencing data

Table S1: Microbiota datasets from qiita

	Sample source	Study ID	Total sample size	Included sample size
1	infant gut	101	63	61
2	infant gut	10293	144	130
3	human and canine gut	10394	1535	1522
4	mice gut	10469	391	321
5	human gut	1561	52	52
6	human gut, HIV	1700	58	58
7	Cape Buffalo gut	1736	642	614
8	human gut	1841	3735	3733
9	human gut, new-onset Crohns disease	1998	284	284
10	human gut, twinsUK population	2014	1081	1024
11	human gut, ICU patients	2136	554	144
12	human gut	455	92	92
13	human gut	457	91	77
14	mice gut	654	212	212
15	human gut, pregnant women	867	1007	772
16	infant gut	10297	85	71
17	monkey gut	10315	199	199
18	grant gazelle gut	10323	768	745
19	human gut, western Oklahoma	10342	58	58
20	human gut	1070	118	114
21	human gut	1189	436	83
22	zebrafish gut	1192	50	47
23	asian primates gut	1453	318	53
24	cow hind gut	1621	192	192
25	mice gut	1634	294	293
26	monkey gut	1696	172	160
27	bat gut	1734	96	94
28	colobine primates gut	2182	167	167
29	human gut	2202	820	534
30	bat gut	2338	192	76
31	human gut	449	602	45
32	human gut	452	160	154
33	human gut	456	158	158
34	human gut	492	77	75
35	human gut (obese and lean twins)	77	281	281
36	human gut	850	528	528
37	freshwater fish gut	940	288	64
38	Iguanas gut	963	100	100
39	human tongue	1248	897	897
40	human hand skin	317	175	175

Table S2: The frequency of 1st rank in the 38 gut microbiome datasets.

	GMPR	CSS	RLE	RLE+	TMM	TMM+	TSS	RAW
OTU(All)	22	7	0	0	0	0	8	1
OTUs(Top)	23	3	1	1	3	0	7	0
OTUs(Middle)	20	8	0	0	1	0	9	0
OTUs(Bottom)	20	8	0	0	2	2	6	0

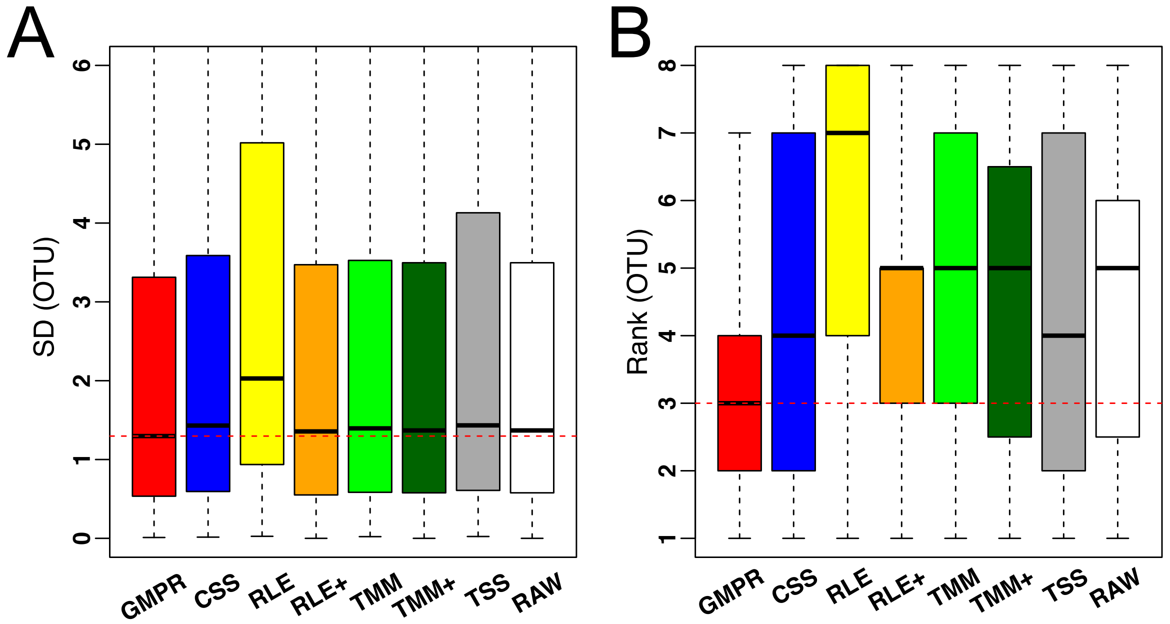

Figure S1: Comparison of normalization methods in reducing inter-sample variability of normalized OTU abundances based on Study 1561. A. Distribution of the standard deviations (SDs) of the normalized OTU abundances for all OTUs. B. Distribution of the ranks of the normalized OTU abundances. Each OTU is ranked based on its SDs among the competing methods.

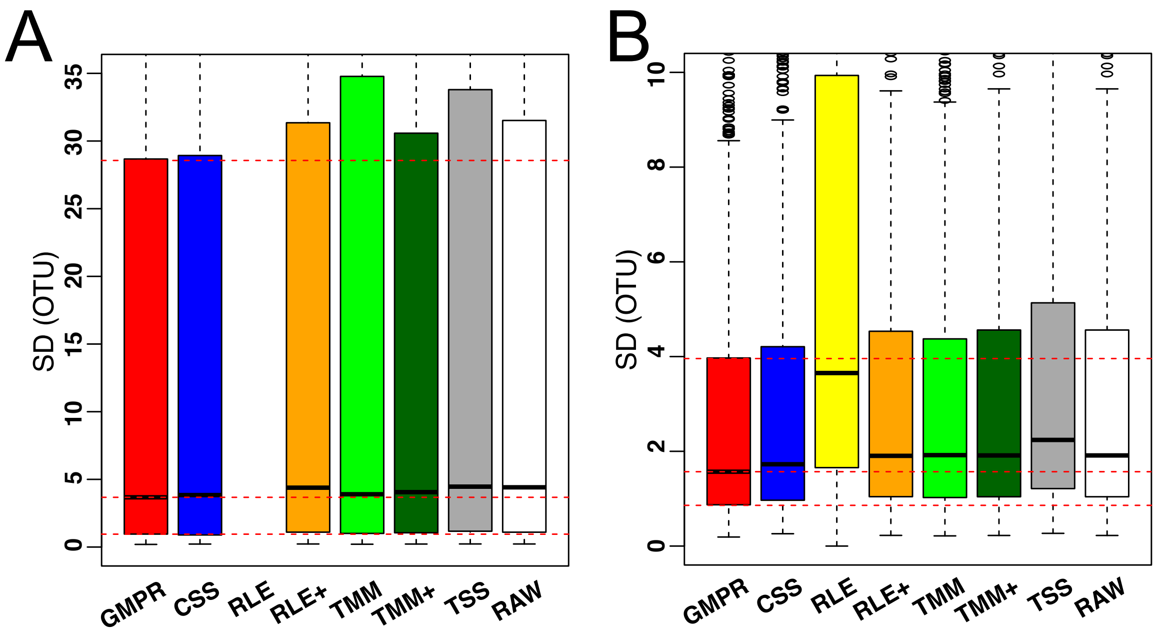

Figure S2: Comparison of normalization methods in reducing inter-sample variability of normalized OTU abundances based on an oral (A) and a skin (B) microbiome dataset. Distribution of the standard deviations (SDs) of the normalized OTU abundances for all OTUs is shown.

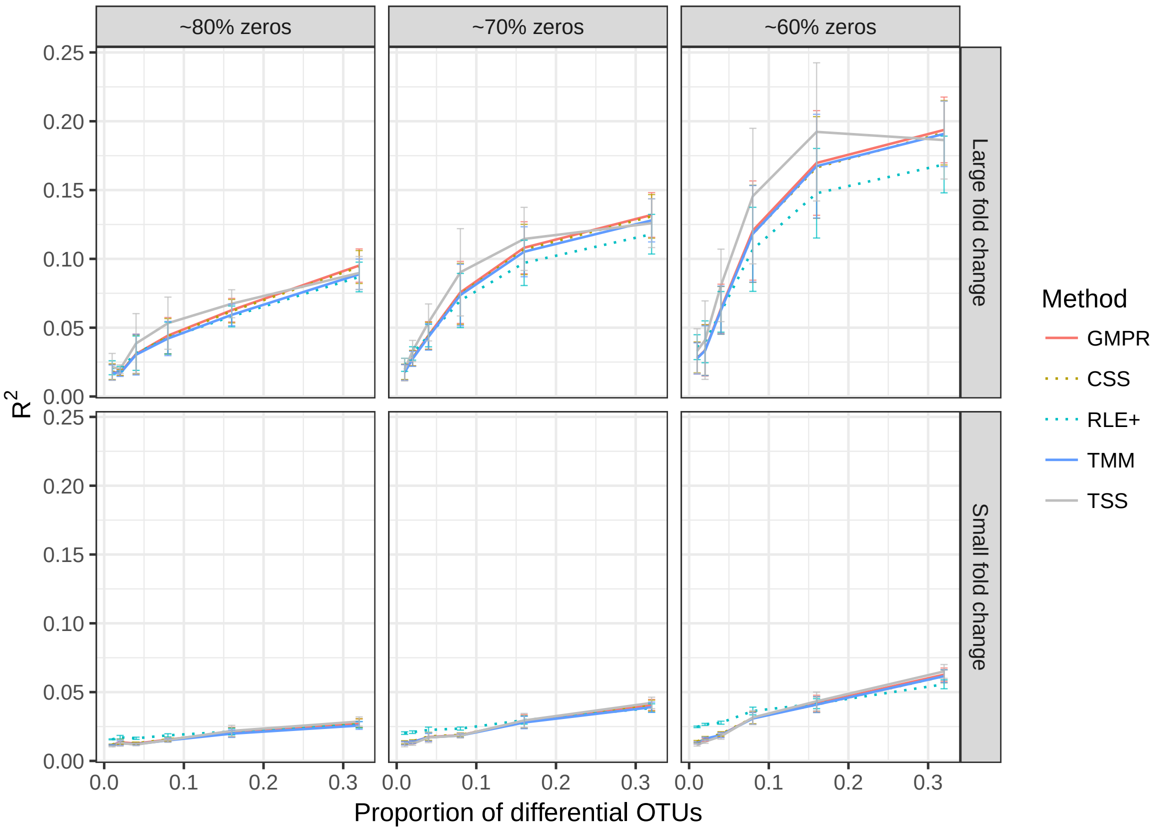

Figure S3: Comparison the performance of different normalization procedures in Bray-Curtis distance-based clustering. Two clusters are simulated with different percentages of differential OTUs using the same simulation strategy as in the "fixed" perturbation simulation (Figure 2). Only half of the samples are applied fold changes to the set of differential OTUs to create two clusters. Counts are normalized using GMPR, CSS, TMM, RLE+ and TSS and Bray-Curtis distances are calculated based on the normalized counts. The clustering performance is assessed using the PERMANOVA R^2 (a large R^2 indicates a clearer separation).
